# Metabolomics reveals early pregnancy biomarkers in sows: a non-invasive diagnostic approach

**DOI:** 10.3389/fvets.2024.1396492

**Published:** 2024-04-25

**Authors:** Yujun Ren, Qingze Zhang, Fan He, Menfan Qi, Binbin Fu, Huapeng Zhang, Tao Huang

**Affiliations:** ^1^College of Animal Science and Technology, Shihezi University, Shihezi, China; ^2^Xinjiang Pig Breeding Engineering Technology Research Center, Xinjiang Tecon Husbandry S&T Co. Ltd, Changji, China

**Keywords:** early pregnancy diagnosis, metabolomics, sows, saliva, biomarker, LC–MS/MS

## Abstract

In an effort to enhance reproductive management and reduce non-productive periods in swine breeding, this study presents a novel, non-invasive metabolomics approach for the identification of early pregnancy biomarkers in sows. Utilizing an untargeted metabolomics approach with mass spectrometry analysis, we examined saliva samples from pregnant (*n* = 6) and non-pregnant control sows (*n* = 6, artificially inseminated with non-viable sperm). Our analysis revealed 286 differentially expressed metabolites, with 152 being up-regulated and 134 down-regulated in the pregnant group. Among these, three metabolites, namely Hyodeoxycholic acid, 2′-deoxyguanosine, and Thymidine, emerged as potential early pregnancy biomarkers. These biomarkers were further evaluated using targeted LC–MS/MS quantification and qualification, accompanied by ROC curve analysis. The study confirmed Hyodeoxycholic acid and 2′-deoxyguanosine as promising biomarkers for early pregnancy detection, offering potential for future implementation in swine production environments. This research establishes a robust theoretical foundation for the development of innovative molecular diagnostic techniques and explores new avenues for molecular genetic breeding and non-invasive diagnostics, ultimately enhancing fertility and productivity in sow herds.

## 1 Introduction

The swine industry is intensifying and scaling, with a focus on reducing non-productive days for sows and minimizing breeding costs. Early gestational diagnosis is crucial for optimizing reproductive management, yet current techniques have limitations (1–3). However, finding an effective strategy to reduce interbirth intervals and decrease non-productive days remains a challenge for the industry (4–6). Early gestational diagnosis plays a crucial role in optimizing reproductive management for sows and contributes favorably to high-quality expansion of pig farming operations (7).

Currently available techniques for pregnancy diagnosis in sows have several limitations. For instance, ultrasound-based diagnosis is prone to user-dependent variability in accuracy and interpretation (8). Outward traits-focused methods, such as detecting abdomen and udder enlargement, often exhibit delayed diagnostic capabilities (9). Hormone-based identification methods can occasionally result in “false estrus” confusions in a small percentage of sows, leading to diagnostic errors (10). Additionally, relying on boar detection for return-to-estrus identification can inadvertently increase the risk of transmitting malignant diseases (11). Currently, B-ultrasound is commonly used for sow pregnancy diagnosis, both domestically and internationally (11). However, this method is not effective in detecting pregnancy during the initial estrus cycle (21 days) after artificial insemination, leading to an increase in non-productive days (12).

In recent years, there has been a growing interest in utilizing salivary biomarkers as a diagnostic tool due to the advantages they offer over traditional methods (13). Saliva collection is non-invasive, easily accessible, and less stressful for animals compared to other sample collection methods such as blood or urine (14). Additionally, saliva contains a wide range of biomolecules, including metabolites, which can provide valuable information about the physiological and metabolic status of an individual (15).

The overarching aim of this research is to identify biomarkers for early pregnancy diagnosis in sows and to assess their applicability in production settings. This is with the expectation of significantly enhancing the efficiency of pregnancy detection in sows, addressing key challenges in production, and fostering the efficient and sustainable development of the swine breeding industry.

## 2 Materials and methods

All experiments involving animals were conducted under a protocol approved by the Medical Ethics Committee, First Affiliated Hospital, Medical College, Shihezi University (2014-073-01, 5 March 2014).

### 2.1 Samples

#### 2.1.1 Laboratory animal handling

Thirty Large White × Yorkshire Binary sows, matched for growth age, parity, body condition, and farrowing status, were enrolled in this study. All sows were in good health and had no genetic diseases. The breeding house temperature was maintained at 18–25°C, with humidity sustained at 50–55%. The production strategy employed an all-in, all-out system, and the sows were fed complete feed and managed daily according to industry regulations. In the experiment, 12 sows were randomly assigned for non-targeted metabolomics research, with 6 designated as the experimental group and the remaining 6 as the control group. The other 18 sows served as a validation group for targeted metabolomics studies. After weaning, estrus identification was carried out twice daily, at 10:00 AM and 4:00 PM, using boars for estrus stimulation. The standing reflex of the sows was used to determine their estrus status, and artificial insemination was performed on the day of estrus detection, defined as day 0 of gestation. Artificial insemination was conducted three times at 12-h intervals. In the experimental group, regular procedures were followed to introduce viable sperm, while in the control group, semen without viable sperm was used [The semen samples were treated by immersing the containers in a water bath maintained at 95°C for 30 min to ensure spermatozoa inactivation. Post-treatment, microscopic analysis confirmed the absence of viable sperm, thereby guaranteeing the non-fertilizing capacity of the semen used in the control group (16)]. On the 18th day post-artificial insemination, boars were again used for estrus stimulation to determine if the sows returned to estrus. On the 25th and 35th day post-artificial insemination, pregnancy was confirmed in the sows using a veterinary B-ultrasound machine.

#### 2.1.2 Sample collection process

Sterile-defatted gauze was utilized as a stimulus to induce chewing behavior in the sows. Subsequently, the thoroughly chewed gauze was collected and saliva was extracted by squeezing it into a centrifuge tube. The centrifuge tube had been pre-treated with high temperature and pressure and was sealed using a self-sealing bag. Once the sample was obtained, it was subjected to centrifugation at 3000 revolutions per minute for a duration of 10 min. After centrifugation, the supernatant, which represents the liquid portion above the sediment, was carefully transferred to a 5 mL cryopreservation tube. To prevent degradation, the cryopreservation tube was then placed in liquid nitrogen for preservation. The preserved samples were transported to the laboratory and stored at a temperature of −80°C until further analysis.

### 2.2 Non-targeted metabolomic analyses

#### 2.2.1 Pre-treatment of non-targeted metabolomics experiments

The experimental procedure was carried out as follows: 1 mL of the sample was passed through a solid-phase extraction (SPE) column, and 3 mL of methanol eluate was collected. The eluate was then dried using a nitrogen blowing instrument. Subsequently, 300 μL of a methanol–water mixture (with a volume ratio of 4:1) containing L-2-chlorophenylalanine at a concentration of 4 μg/mL was added to the dried extract. The mixture was vortexed for 1 min and then subjected to ultrasonication in an ice-water bath for 10 min. The sample was then left undisturbed at −40°C for 30 min and subsequently centrifuged at 13,000 rpm for 10 min at 4°C. A total of 150 μL of the supernatant from the third extraction was pipetted and passed through a 0.22 μm filter before being transferred to an LC injection vial. The vial was then stored in a refrigerator at −80°C for further analysis. The same process was repeated for the quality control samples, which were prepared by mixing equal volumes of sample extracts from the pregnancy group and the non-pregnancy group.

#### 2.2.2 Non-targeted metabolomics liquid chromatography-mass spectrometry analysis conditions

Chromatographic conditions: ACQUITY UPLC HSS T3 was used with a column (100 mm × 2.1 mm, 1.8 um); the column temperature was maintained at 45°C; the mobile phases were A-water (containing 0.1% formic acid) and B-acetonitrile; the flow rate was maintained at 0.35 mL/min; the injection volume was 2 μL.

Mass spectrometry conditions: the ion source was ESI; positive and negative ion scanning modes were used for signal acquisition.

### 2.3 Targeted metabolomic profiling

#### 2.3.1 Sample pre-treatment

After thawing the samples, they were vortexed for 10 s and thoroughly mixed. Then, 50 μL of the samples were transferred to numbered 1.5 mL centrifuge tubes and immediately stored at −80°C. Subsequently, 250 μL of a 20% acetonitrile-methanol extraction solution was added to each tube. The samples were vortexed for 3 min and centrifuged at 12,000 r/min for 10 min at 4°C. The resulting supernatant was carefully aspirated and transferred to another numbered 1.5 mL centrifuge tube, which was then placed in a refrigerator at −20°C for 30 min. Following this, 250 μL of the supernatant was extracted into another centrifuge tube and further centrifuged for 30 min at −20°C, followed by an additional centrifugation at 12,000 r/min and 4°C for 10 min. From the resulting supernatant, 180 μL was passed through a protein precipitation plate for analysis, with the remaining supernatant stored at −20°C in the refrigerator.

#### 2.3.2 Chromatography and mass spectrometry acquisition conditions

The data acquisition instrumentation consisted primarily of Ultra Performance Liquid Chromatography (UPLC) (ExionLC™ AD)^1^ and Tandem Mass Spectrometry (MS/MS) (QTRAP® 6,500+, see text footnote 1). The T3 method under liquid phase conditions mainly includes: Chromatographic column: Waters ACQUITY UPLC HSS T3 C18 column (1.8 μm, 100 mm × 2.1 mm i.d.); Mobile phase: A phase, ultrapure water (containing 0.05% formic acid); B phase, acetonitrile (containing 0.05% formic acid); Flow rate of 0.35 mL/min; column temperature of 40°C; injection volume of 2 μL; Gradient elution: 0 min A/B ratio of 95:5 (V/V), 8 min A/B ratio of 5:95 (V/V), 9.5 min A/B ratio of 5:95 (V/V), 9.6 min A/B ratio of 95:5 (V/V), 12 min A/B ratio of 95:5 (V/V).

The Amide method under liquid phase conditions mainly includes: Chromatographic column: ACQUITY UPLC BEH Amide column (1.7 μm, 100 mm × 2.1 mm i.d.); Mobile phase: A phase, ultrapure water (10 mM ammonium acetate, 0.3% ammonia); B phase, 90% acetonitrile/water (V/V); Flow rate of 0.40 mL/min; column temperature of 40°C; injection volume of 2 μL; Gradient elution: 0–1.2 min A/B ratio of 5:95 (V/V), 8 min A/B ratio of 30:70 (V/V), 9.0–11 min A/B ratio of 50:50 (V/V), 11.1–15 min A/B ratio of 5:95 (V/V).

The mass spectrometry conditions mainly include: Electrospray ionization (ESI) temperature of 550°C, mass spectrometry voltage of 5,500 V in positive ion mode, mass spectrometry voltage of −4,500 V in negative ion mode, curtain gas (CUR) pressure of 35 psi. In the Q-Trap 6,500+, the ion pairs are scanned and detected based on the optimized declustering potential (DP) and collision energy (CE).

### 2.4 Statistical analysis

In this study, unsupervised Principal Component Analysis (PCA) was utilized to analyze the overall distribution and stability of the data in both the pregnancy and non-pregnancy groups. The primary parameter of the PCA model was R2X. The PCA score plot, with t [1] on the *x*-axis and t [2] on the *y*-axis, represents the projected scores of each sample onto the first two principal components, PC1 and PC2, respectively. This approach allows for a comprehensive assessment of the data without any prior assumptions or bias. After applying unsupervised PCA, candidate metabolites were selected based on their expression patterns and confidence. The Variable Important in Projection (VIP) values, which indicate the importance of each metabolite in the projection, were used as a measure of confidence. Metabolites with high VIP values were considered more reliable and were further analyzed in the subsequent steps. The differential metabolites identified in the analysis were then explored for their potential involvement in pathway and signaling analyses. This step provides insights into the underlying biological mechanisms associated with pregnancy and non-pregnancy.

To confirm the statistical significance of the observed differences between the pregnancy and non-pregnancy groups, *t*-tests were performed. The between-group variability, as revealed by the *t*-tests, supports the validity of the observed differences. A volcano plot was utilized for the visualization of *p*-values and fold change (FC) values. The *x*-axis represents the log2-transformed fold change (log2FC) values derived from the comparison between two groups, while the *y*-axis depicts the negative logarithm (base 10) of the *p*-values (−log10 [*p*-value]). Data points colored in red indicate metabolites with a *p*-value less than 0.05 and an FC greater than 1, signifying statistically significant upregulation of these metabolites. Data points in blue denote metabolites with a *p*-value less than 0.05 and an FC less than 1, indicating statistically significant downregulation. In order to assess the diagnostic performance of the identified metabolites and their combination as biomarkers, Receiver Operating Characteristic (ROC) analysis was conducted. ROC curves provide a graphical representation of the sensitivity and specificity of the diagnostic tests. The AUC, a summary measure of the overall diagnostic accuracy, was calculated from the ROC curve.

## 3 Results

Sows participating in the non-targeted metabolism study were evaluated using ultrasound on days 25 and 35 after breeding. In the normal insemination group, all sows were found to be pregnant, while in the control group, none of the sows were pregnant. In the targeted metabolism study, sows were examined by ultrasound on days 25 and 35 after mating. Out of the sows examined, 5 were determined to be non-pregnant, while 12 were confirmed to be pregnant.

### 3.1 Based On LC–MS untargeted metabolomic results

#### 3.1.1 Quality control analysis

**Figure 1:**
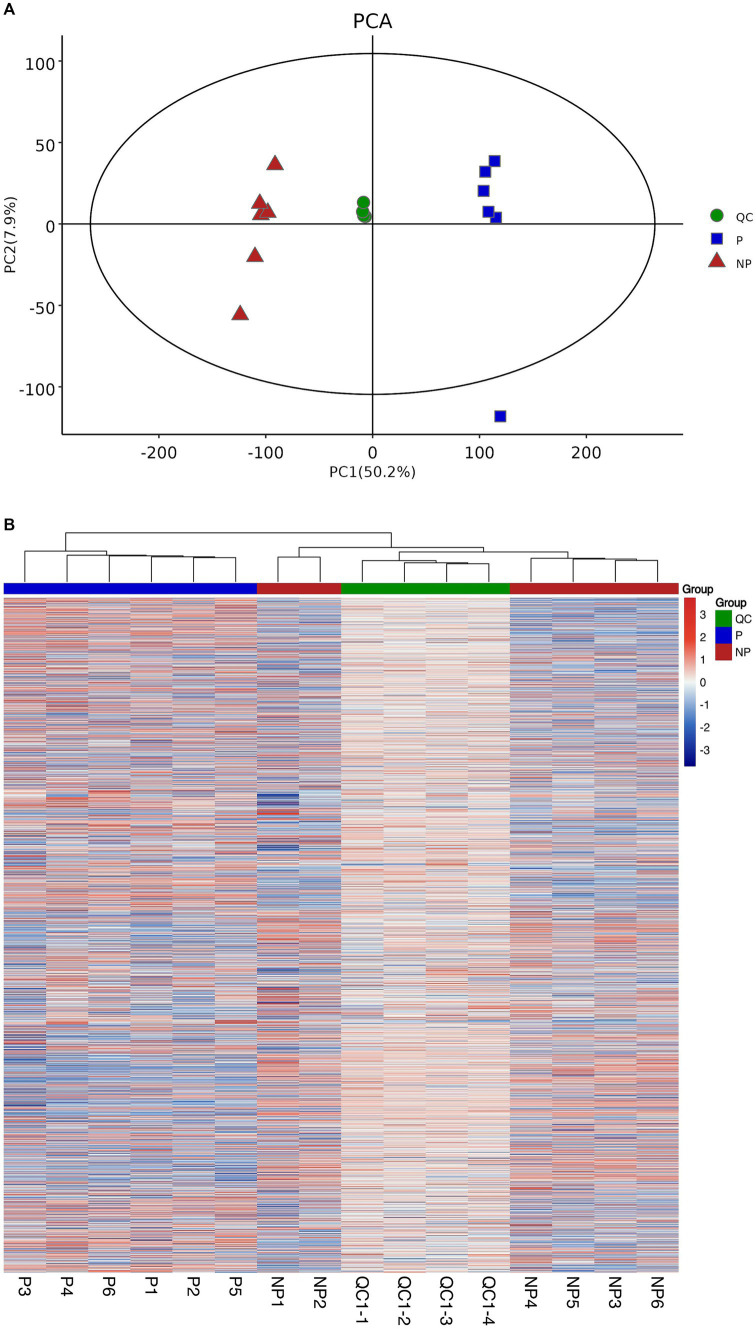
Sample quality control analyses. (A) Graph of PCA scores for pregnancy, non-pregnancy and quality control groups. (B) Heatmap of clustering of pregnancy, non-pregnancy and quality control groups.

To assess the stability of the mass spectrometry system, we employed the PCA method. The PCA model plots, derived from 7-fold cross-validation, are presented in Figure 1A. The QC samples are observed to be tightly clustered in the PCA model, which is a strong indication of the instrumental detection’s consistency throughout the experimental process. This clustering pattern suggests that the mass spectrometry system maintained a high level of stability, which is crucial for obtaining accurate and reproducible results.

Furthermore, to elucidate the relationship between the QC samples and the remaining samples, as well as to reinforce the stability of the QC samples, a hierarchical cluster analysis was conducted on all metabolite expressions. The dendrogram resulting from this analysis is depicted in Figure 1B, where the QC samples are clearly shown to be consistent with each other, further validating their reliability.

#### 3.1.2 Differential metabolite analysis

**Figure 2:**
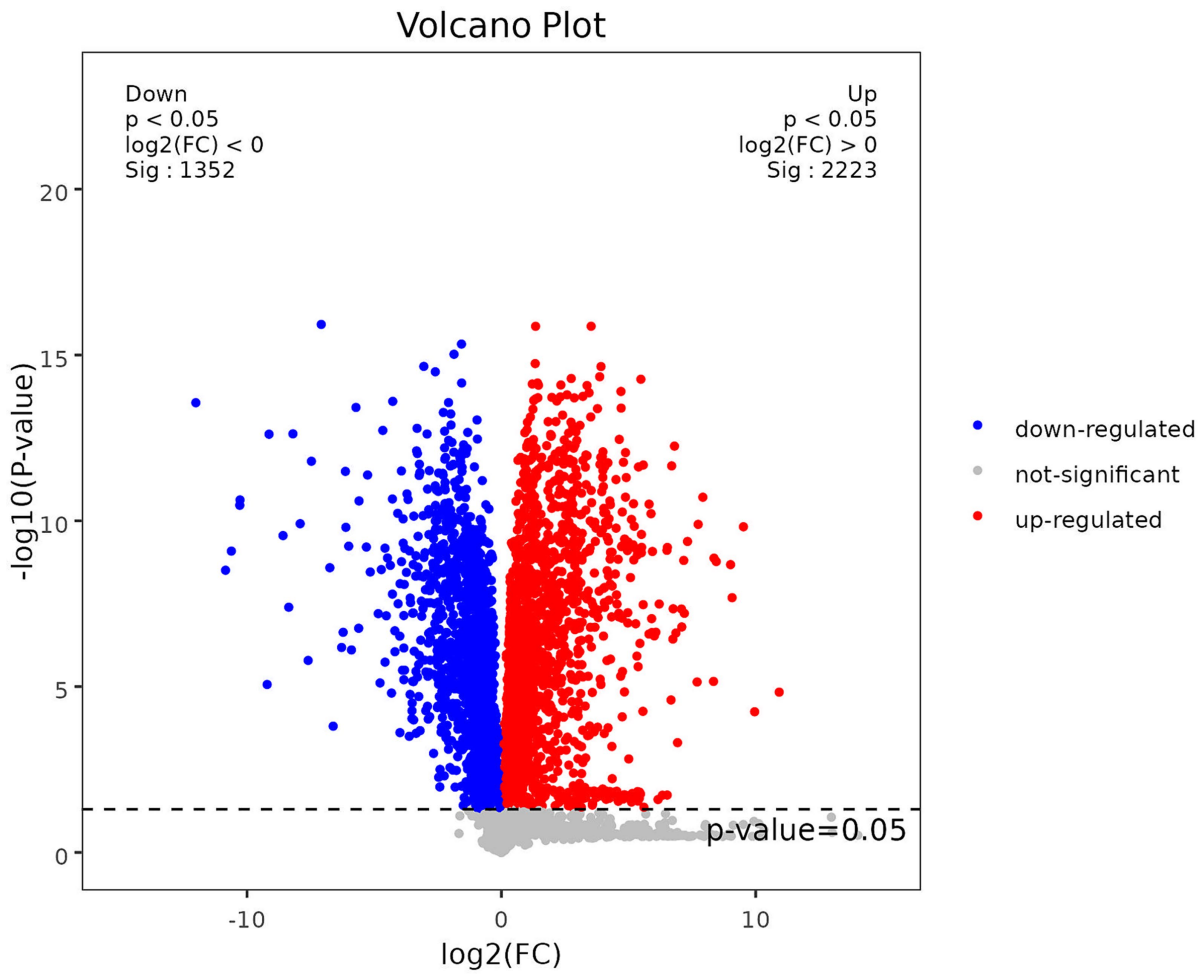
Volcano plot of differential metabolites.

In order to quantify the number of differential metabolites in each comparison group, we counted the number of differential metabolites. A total of 286 differential metabolites were identified, of which 152 were up-regulated and 134 were down-regulated compared to the control group. To visually analyze the metabolite *p*-values and FC-values obtained from mass spectrometry analysis, a volcano diagram was employed to screen the differential metabolites, as illustrated in Figure 2.

**Figure 3:**
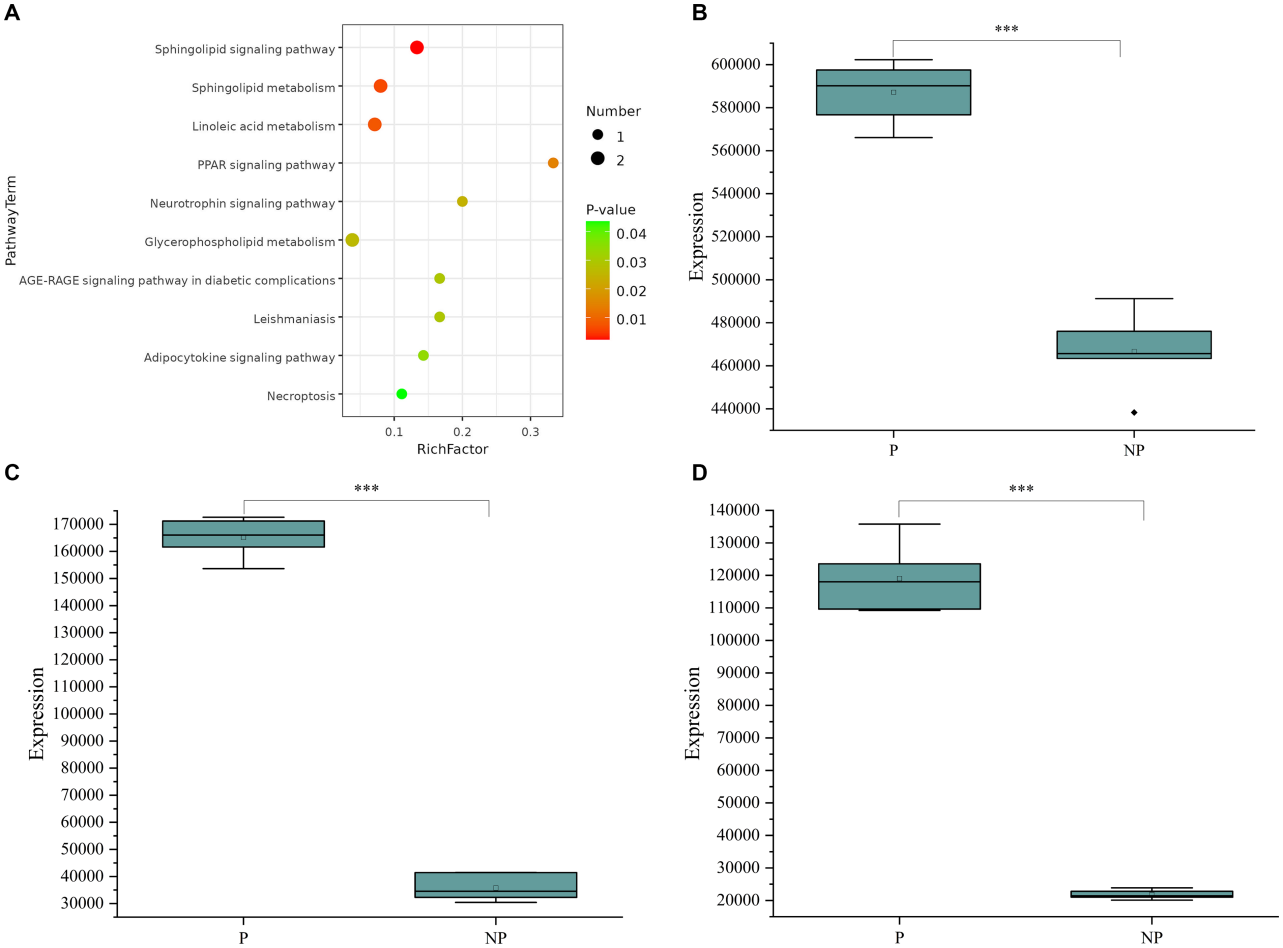
(A) Metabolic pathway bubble chart. (B) Boxplot of Hyodeoxycholic acid expression in pregnant and non-pregnant sows. (C) Box line plot of 2’-deoxyguanosine expression in pregnant and non-pregnant sows. (D) Boxplot of Thymidine expression in pregnant and non-pregnant sows. The data (*n* = 12) were analyzed by Student’s t-test. *P < 0.05, **P < 0.01, ***P < 0.001.

To delve deeper into the biological implications of the differential metabolites, we conducted a KEGG (Kyoto Encyclopedia of Genes and Genomes) pathway analysis, as illustrated in Figure 3A. The enrichment analysis highlighted that the differentially expressed metabolites were primarily associated with pathways such as the Sphingolipid signaling pathway, Sphingolipid metabolism, and Linoleic acid metabolism. By integrating the results of the enrichment analysis with the VIP values of the metabolites, we identified three potential biomarkers for sow pregnancy diagnosis: Hyodeoxycholic acid, 2′-deoxyguanosine, and Thymidine. These metabolites were chosen based on their significant expression levels and their potential roles in the biological processes related to pregnancy. To further investigate the expression levels of the identified biomarkers, we generated box plots for each, as shown in Figures 3B–D. Box plots provide a detailed view of the distribution of the metabolite levels, including the median, quartiles, and outliers, which is essential for evaluating the potential of these biomarkers for diagnostic purposes.

### 3.2 Based on LC–MS/MS targeted metabolomic results

#### 3.2.1 Quality control analysis

**Figure 4:**
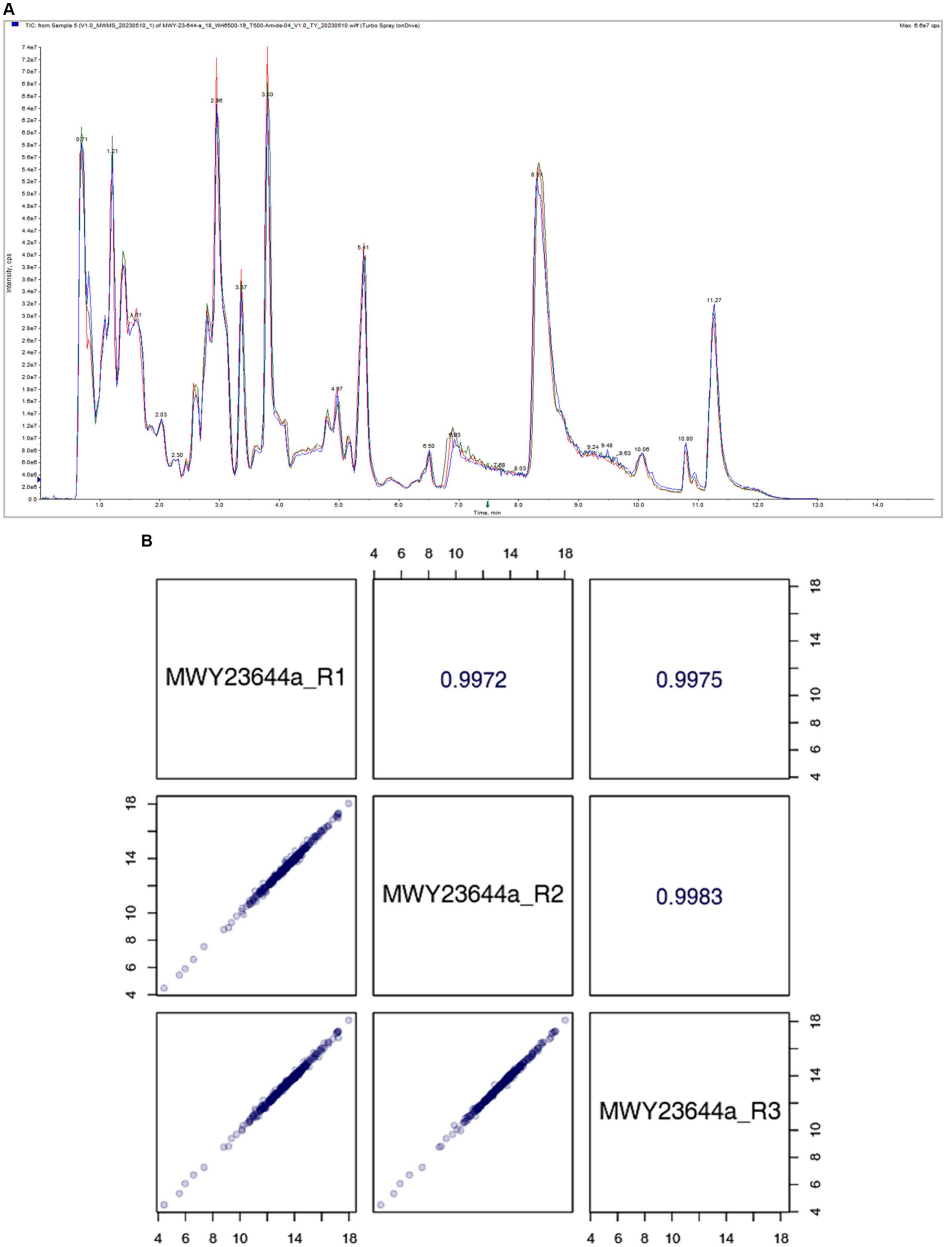
(A) Total Ion Chromatogram. (B) Quality Control (QC) sample correlation analysis.

The stability of the mass spectrometry signal was assessed by analyzing the total ion flow for metabolite detection. A high degree of curve overlap in the retention time and peak intensity indicates that the mass spectrometry system maintained good signal stability even when the same sample was detected at different time points. This consistency in signal detection is crucial for ensuring the reliability and reproducibility of the metabolite profiling, as depicted in Figure 4A.

The correlation scatter plot in the lower-left diagonal square of Figure 4B represents the metabolite content of each point, with the horizontal and vertical coordinates corresponding to the levels of metabolites detected in the QC samples. The close correlation between the metabolite contents across multiple runs indicates the precision and consistency of the mass spectrometry system in detecting and quantifying metabolites. The upper-right diagonal square in Figure 4B displays the correlation coefficient for the corresponding QC sample. A high correlation coefficient signifies a strong agreement between repeated measurements, further reinforcing the reliability of the mass spectrometry data.

#### 3.2.2 Analysis of the diagnostic effectiveness of screening biomarkers

In this study, we evaluated the diagnostic effectiveness of three potential biomarkers, Hyodeoxycholic acid, 2′-deoxyguanosine, and Thymidine, for detecting pregnancy in sows. These biomarkers were quantitatively analyzed using a liquid chromatography–tandem mass spectrometry (LC–MS/MS) detection platform, and their concentrations in saliva samples from pregnant and non-pregnant sows were compared.

**Figure 5:**
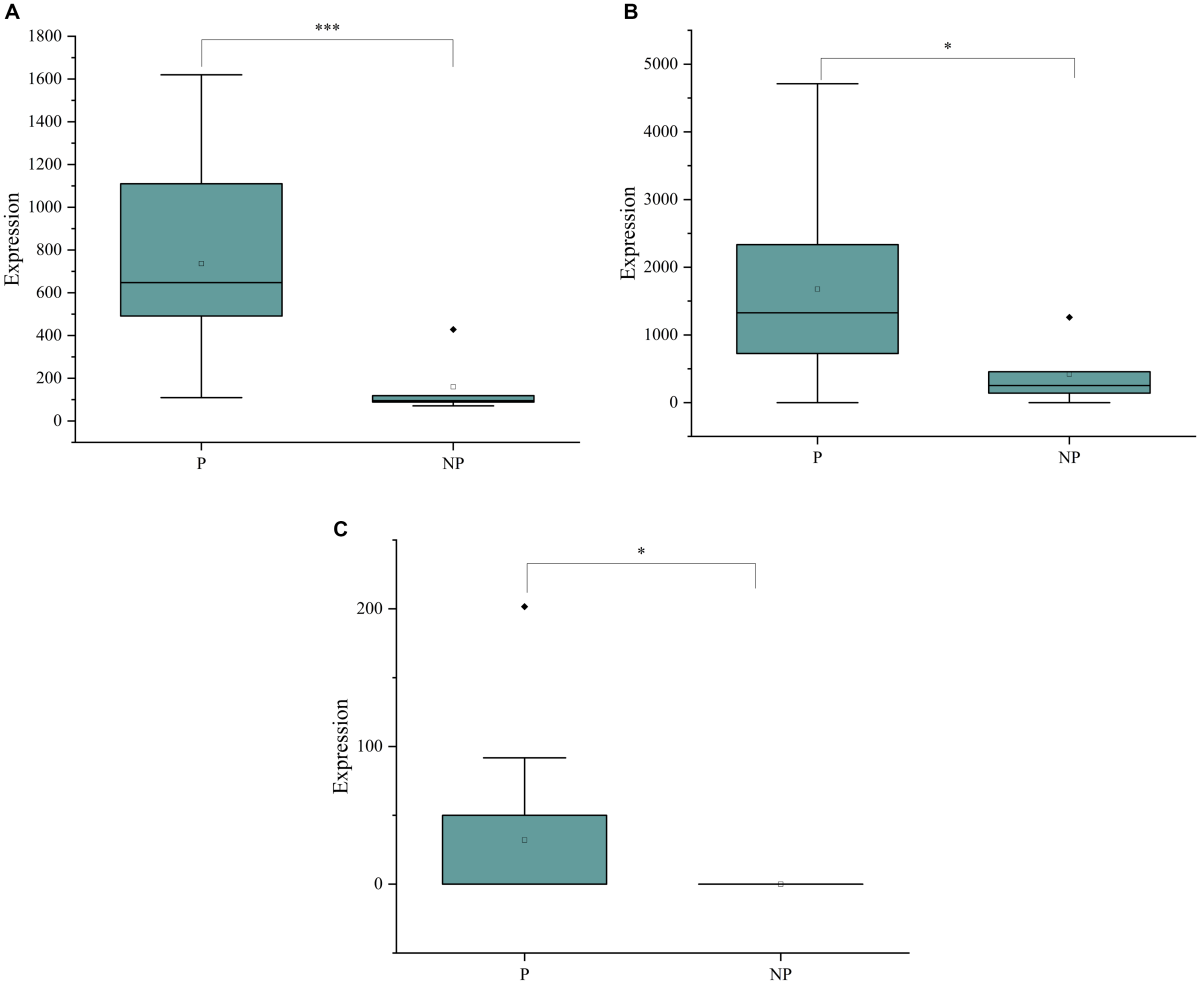
(A) Boxplots of Hyodeoxycholic acid concentrations in the saliva of pregnant and non-pregnant sows. (B) Boxplots of 2’-deoxyguanosine concentrations in the saliva of pregnant and non-pregnant sows. (C) Boxplots of Thymidine concentrations in the saliva of pregnant and non-pregnant sows.

- The boxplot in Figure 5A shows the concentrations of Hyodeoxycholic acid in saliva samples. A significant difference in the levels of this metabolite was observed between the pregnant and non-pregnant groups, with *p*-values less than 0.05, indicating its potential as a diagnostic biomarker for pregnancy.
- Similarly, Figure 5B presents the boxplot for 2′-deoxyguanosine concentrations. The analysis revealed a significant difference in the levels of this biomarker between the two groups, further supporting its potential role in pregnancy diagnosis.
- The boxplot in Figure 5C illustrates the concentrations of Thymidine in saliva. A significant difference was also observed for this metabolite between pregnant and non-pregnant sows, suggesting its utility as a biomarker for detecting pregnancy.

To ensure the accuracy of the biomarker detection, standard curves were generated, and their linear equations are detailed in Table 1. These curves are essential for the quantification of the biomarkers and validation of the assay. The significant differences in the levels of these metabolites between the pregnant and non-pregnant groups, as confirmed by the t-test analysis, underscore the potential of Hyodeoxycholic acid, 2′-deoxyguanosine, and Thymidine as diagnostic biomarkers. The detailed standard curve data further validate the robustness of the LC–MS/MS detection platform used in this study.

**Table 1:** Table of linear equations for standard curves for biomarker detection.

Index	Class	RT	Equation	*r*	Weighting	LLOQ	ULOQ
Glycohyodeoxycholic-acid	Bile acids	6.02	*y* = 11890.54193 x + 13559.86456	0.99438	1/x	2	500
Thymidine	Nucleotide and its metabolomics	2.06	*y* = 310.19354 x + 23597.76294	0.99413	1/x	20	500
2′-deoxyguanosine	Nucleotide and its metabolomics	2.29	*y* = 597.46303 x + 10553.98313	0.99197	1/x	100	1,000

### 3.3 Performance of the early pregnancy diagnosis model

The early pregnancy diagnosis model developed in this study was evaluated using Receiver Operating Characteristic (ROC) curves, a standard tool for assessing the diagnostic performance of biomarkers. The area under the curve (AUC) is a key metric for evaluating the diagnostic ability of the biomarkers, with higher AUC values indicating superior diagnostic performance.

**Figure 6:**
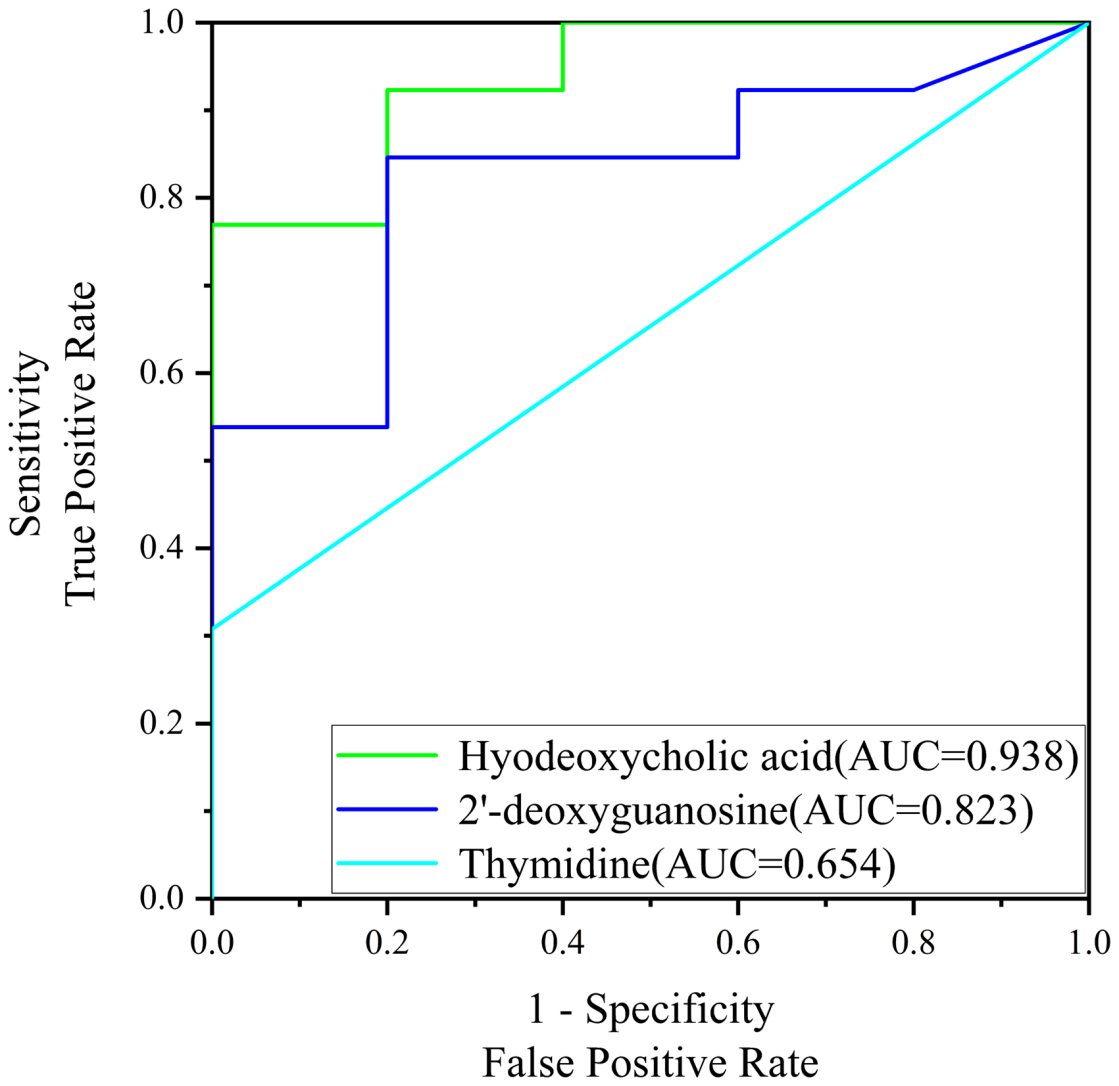
ROC curve graph for the screening of biomarkers.

Figure 6 presents the ROC curves for the screening biomarkers in this study. For Hyodeoxycholic acid, the AUC was determined to be 0.938, which is indicative of its strong diagnostic value and suggests a high potential for practical application in diagnostics and therapeutics. This high AUC value reflects the biomarker’s ability to accurately distinguish between pregnant and non-pregnant sows with a high degree of sensitivity and specificity. Similarly, 2′-deoxyguanosine exhibited an AUC of 0.823, which, although lower than Hyodeoxycholic acid, still indicates a significant diagnostic value. This value suggests that 2′-deoxyguanosine could be a useful biomarker for early pregnancy diagnosis, contributing to the development of diagnostic tools with good accuracy. However, Thymidine showed a lower diagnostic AUC of 0.654, which suggests that its diagnostic potential is less robust compared to the other two biomarkers. Despite this, it may still offer some value in combination with other biomarkers as part of a panel test for early pregnancy detection. In conclusion, the ROC curve analysis demonstrates the diagnostic potential of Hyodeoxycholic acid and 2′-deoxyguanosine as biomarkers for early pregnancy diagnosis in sows.

## 4 Discussion

The current study has successfully harnessed the non-invasive potential of salivary metabolomics to identify key biomarkers in early pregnant sows. Our approach has not only provided a novel perspective on swine reproductive management but also laid the groundwork for future molecular diagnostic techniques. The use of saliva as a biological sample is advantageous due to its minimally invasive nature, ease of collection, and the comprehensive metabolic data it offers (17–20). The rich vascularization of salivary glands facilitates an efficient exchange of metabolites, making saliva an ideal matrix for reflecting systemic metabolic changes (19, 20).

Through our untargeted metabolomics analysis, we identified 286 differentially expressed metabolites, with Hyodeoxycholic acid and 2′-deoxyguanosine showing significant differential expression and high diagnostic accuracy as potential biomarkers for early pregnancy. The validation of these biomarkers using LC–MS/MS confirmed their high diagnostic value, which is expected to contribute to a paradigm shift in the swine industry’s reproductive management strategies.

Our findings are in line with other studies that have utilized salivary metabolomics to detect changes in metabolic pathways associated with various physiological conditions (10, 21–23). In the context of swine reproduction, the development of early pregnancy biomarkers is of paramount importance. Traditional methods, such as real-time ultrasound (RTU) imaging, are limited by their timing constraints and the need for specialized equipment and expertise (7, 24, 25). By contrast, our non-invasive metabolomic approach offers a promising alternative that can be implemented earlier in the gestation period, potentially reducing non-productive days and improving herd management.

The high AUC values for Hyodeoxycholic acid (0.938) and 2′-deoxyguanosine (0.823) underscore their potential as robust biomarkers for early pregnancy detection. Hyodeoxycholic acid, in particular, has been recognized for its role in lipid metabolism, and its up-regulation may reflect the metabolic adjustments associated with early pregnancy (26, 27). In the context of early pregnancy diagnosis in sows, our study has identified elevated levels of Hyodeoxycholic acid in the saliva of pregnant animals, which presents a novel perspective for non-invasive diagnostic approaches. The up-regulation of 2′-deoxyguanosine during early pregnancy may reflect the increased demand for nucleotides required for the rapid cellular proliferation and growth that occurs in the early stages of embryonic development. 2′-deoxyguanosine, a nucleoside analog, is known for its role in nucleic acid metabolism and has been implicated in various biological processes, including DNA replication and repair, which is integral to nucleic acid metabolism, suggests a connection between early gestational metabolic changes and nucleotide requirements for embryonic development (28–30). However, the precise mechanisms underlying the association between Hyodeoxycholic acid and 2′-deoxyguanosine levels and early pregnancy in sows remain to be elucidated. Future research should focus on the functional analysis of this biomarker within the broader metabolic landscape, as well as the validation of its diagnostic efficacy across different breeds and under various environmental conditions. Additionally, the potential of Hyodeoxycholic acid and 2′-deoxyguanosine as a prognostic indicator for pregnancy outcomes, such as litter size and health, warrants further investigation.

Despite the promising results, there are limitations to our study that necessitate further research. Firstly, the sample size was relatively small, which may limit the generalizability of our findings. Future studies should include a larger number of sows to validate the identified biomarkers. Secondly, while saliva has been the focus of our study, it would be beneficial to explore other biological samples such as blood or urine to corroborate the reliability of these metabolites as pregnancy markers (31). Thirdly, the biological functions of these metabolites during pregnancy and their potential as prognostic indicators for pregnancy outcomes require further investigation, potentially incorporating gene expression and proteomics data (32, 33).

In conclusion, our study represents a significant step forward in the development of innovative, non-invasive diagnostic techniques for early pregnancy detection in sows. The identified biomarkers, Hyodeoxycholic acid and 2′-deoxyguanosine, hold promise for improving reproductive management practices, ultimately enhancing productivity and welfare in swine breeding operations. Future research should focus on validating these biomarkers in larger cohorts and exploring their functional roles in the context of pregnancy, paving the way for their practical application in the swine industry.

## 5 Conclusion

In this investigation, we have adeptly employed metabolomics to identify novel biomarkers for early pregnancy detection in sows, focusing on Hyodeoxycholic acid and 2′-deoxyguanosine. Leveraging saliva as a non-invasive substrate, we have unraveled a comprehensive metabolic profile, offering innovative insights into swine reproductive management. The prospective incorporation of these biomarkers into routine health assessments may instigate a transformative change in reproductive strategies. Such advancements have the potential to markedly augment productivity and confer substantial economic benefits to producers, aligning with the industry’s quest for sophisticated production optimization solutions.

## Data availability statement

The raw data supporting the conclusions of this article will be made available by the authors, without undue reservation.

## Ethics statement

The animal studies were approved by the Medical Ethics Committee, First Affiliated Hospital, Medical College, Shihezi University. The studies were conducted in accordance with the local legislation and institutional requirements. Written informed consent was obtained from the owners for the participation of their animals in this study.

## Author contributions

YR: Conceptualization, Data curation, Formal analysis, Investigation, Methodology, Project administration, Software, Supervision, Validation, Visualization, Writing – original draft, Writing – review & editing. QZ: Writing – review & editing, Data curation, Formal analysis. FH: Writing – review & editing, Investigation, Resources, Validation. MQ: Investigation, Validation, Writing – review & editing, Resources. BF: Data curation, Writing – review & editing. HZ: Writing – review & editing, Data curation. TH: Funding acquisition, Methodology, Project administration, Writing – review & editing.

## Footnotes

^1^ https://sciex.com.cn/

## References

[1] Wang G Wang J Chen S Zhao CE. Vertical integration selection of Chinese pig industry chain under African swine fever - from the perspective of stable pig supply. PLoS One. (2023) 18:e0280626. doi: 10.1371/journal.pone.0280626, PMID: 36821546 PMC9949627

[2] Zhao Q Tao C Pan J Wei Q Zhu Z Wang L. Equine chorionic gonadotropin pretreatment 15 days before fixed-time artificial insemination improves the reproductive performance of replacement gilts. Animal. (2021) 15:100406. doi: 10.1016/j.animal.2021.100406 34844186

[3] Koketsu Y Iida R. Farm data analysis for lifetime performance components of sows and their predictors in breeding herds. Porcine Health Manag. (2020) 6:24. doi: 10.1186/s40813-020-00163-1, PMID: 32963803 PMC7499956

[4] Zhou C Cai G Meng F Xu Z He Y Hu Q. Deep-sequencing identification of MicroRNA biomarkers in serum exosomes for early pig pregnancy. Front Genet. (2020) 11:536. doi: 10.3389/fgene.2020.00536, PMID: 32528535 PMC7264423

[5] Tani S Piñeiro C Koketsu Y. High-performing farms exploit reproductive potential of high and low prolific sows better than low-performing farms. Porcine Health Manag. (2018) 4:15. doi: 10.1186/s40813-018-0091-8, PMID: 30026960 PMC6047137

[6] Koketsu Y Tani S Iida R. Factors for improving reproductive performance of sows and herd productivity in commercial breeding herds. Porcine Health Manag. (2017) 3:1. doi: 10.1186/s40813-016-0049-7, PMID: 28405457 PMC5382409

[7] Gokuldas PP Shinde KR Naik S Sahu AR Singh SK Chakurkar EB. Assessment of diagnostic accuracy and effectiveness of trans-abdominal real-time ultrasound imaging for pregnancy diagnosis in breeding sows under intensive management. Trop Anim Health Prod. (2023) 55:239. doi: 10.1007/s11250-023-03649-6, PMID: 37326691

[8] Williams SI Piñeyro P de la Sota RL. Accuracy of pregnancy diagnosis in swine by ultrasonography. Can Vet J. (2008) 49:269–73. PMID: 18390099 PMC2249720

[9] Chae JW Choi YH Lee JN Park HJ Jeong YD Cho ES. An intelligent method for pregnancy diagnosis in breeding sows according to ultrasonography algorithms. J Anim Sci Technol. (2023) 65:365–76. doi: 10.5187/jast.2022.e107, PMID: 37093914 PMC10119456

[10] Liu X Schwarz T Murawski M Tayade C Kridli R Prieto Granados AM. Measurements of circulating progesterone and estrone sulfate concentrations as a diagnostic and prognostic tool in porcine pregnancy revisited. Domest Anim Endocrinol. (2020) 71:106402. doi: 10.1016/j.domaniend.2019.106402, PMID: 31972516

[11] Am-in N Tantasuparuk W Techakumphu M. Comparison of artificial insemination with natural mating on smallholder farms in Thailand, and the effects of boar stimulation and distance of semen delivery on sow reproductive performance. Trop Anim Health Prod. (2010) 42:921–4. doi: 10.1007/s11250-009-9508-3, PMID: 20012195

[12] Tani S Piñeiro C Koketsu Y. Characteristics and risk factors for severe repeat-breeder female pigs and their lifetime performance in commercial breeding herds. Porcine Health Manag. (2017) 3:12. doi: 10.1186/s40813-017-0059-0, PMID: 28603642 PMC5463492

[13] Khijmatgar S Yong J Rübsamen N Lorusso F Rai P Cenzato N. Salivary biomarkers for early detection of oral squamous cell carcinoma (OSCC) and head/neck squamous cell carcinoma (HNSCC): a systematic review and network meta-analysis. Jpn Dent Sci Rev. (2024) 60:32–9. doi: 10.1016/j.jdsr.2023.10.003, PMID: 38204964 PMC10776379

[14] Kumar P Gupta S Das BC. Saliva as a potential non-invasive liquid biopsy for early and easy diagnosis/prognosis of head and neck cancer. Transl Oncol. (2024) 40:101827. doi: 10.1016/j.tranon.2023.101827 38042138 PMC10701368

[15] Kashyap B Hyvärinen E Laitinen I Kullaa AM. Salivary metabolomics in patients with oral lichen planus: a preliminary study based on NMR spectroscopy. Clin Oral Investig. (2024) 28:103. doi: 10.1007/s00784-023-05389-1, PMID: 38236502 PMC10796579

[16] Chao SB Guo L Ou XH Luo M Wang B Schatten H. Heated spermatozoa: effects on embryonic development and epigenetics. Hum Reprod. (2012) 27:1016–24. doi: 10.1093/humrep/des005, PMID: 22313867

[17] Jiang X Chen X Wang T Li Y Pan A Wu J. Perfluorinated polymer modified vertical silicon nanowires as ultra low noise laser desorption ionization substrate for salivary metabolites profiling. Talanta. (2021) 225:122022. doi: 10.1016/j.talanta.2020.122022, PMID: 33592752

[18] Kajiwara N Kakihana M Maeda J Kaneko M Ota S Enomoto A. Salivary metabolomic biomarkers for non-invasive lung cancer detection. Cancer Sci. (2024) 28. doi: 10.1111/cas.16112, PMID: 38417449 PMC11093188

[19] Li Y Guan C Liu C Li Z Han G. Disease diagnosis and application analysis of molecularly imprinted polymers (MIPs) in saliva detection. Talanta. (2024) 269:125394. doi: 10.1016/j.talanta.2023.125394 37980173

[20] Ciurli A Mohammed Y Ammon C Derks RJE Olivier-Jimenez D Ducarmon QR. Spatially and temporally resolved metabolome of the human oral cavity. iScience. (2024) 27:108884. doi: 10.1016/j.isci.2024.108884 38318352 PMC10839270

[21] Moreau C El Habnouni C Lecron JC Morel F Delwail A Le Gall-Ianotto C. Salivary metabolome indicates a shift in tyrosine metabolism in patients with burning mouth syndrome: a prospective case-control study. Pain. (2023) 164:e144–56. doi: 10.1097/j.pain.0000000000002733, PMID: 35916738

[22] Goudet G Nadal-Desbarats L Douet C Savoie J Staub C Venturi E. Salivary and urinary metabolome analysis for pre-puberty-related biomarkers identification in porcine. Animal. (2019) 13:760–70. doi: 10.1017/S1751731118002161, PMID: 30182861 PMC6425368

[23] Fletcher L Akhtar N Zhan X Jafarikia M Sullivan BP Huber LA. Identification of candidate salivary, urinary and serum metabolic biomarkers for high litter size potential in sows (*Sus scrofa*). Meta. (2022) 12:1045. doi: 10.3390/metabo12111045, PMID: 36355128 PMC9697495

[24] Ohtaki T Moriyoshi M Nakada K Nakao T Kawata K. Radioimmunoassay of saliva estrone sulfate in pregnant sows. J Vet Med Sci. (1997) 59:759–63. doi: 10.1292/jvms.59.759, PMID: 9342698

[25] Kousenidis K Giantsis IA Karageorgiou E Avdi M. Swine ultrasonography numerical modeling for pregnancy diagnosis and prediction of litter size. Int J Biol Biomed Eng. (2021) 15:29–35. doi: 10.46300/91011.2021.15.5

[26] Pan J Wu J Zhang S Wang K Ji G Zhou W. Targeted metabolomics revealed the mechanisms underlying the role of Liansu capsule in ameliorating functional dyspepsia. J Ethnopharmacol. (2024) 321:117568. doi: 10.1016/j.jep.2023.117568, PMID: 38092317

[27] Wang P Chen Q Yuan P Lin S Chen H Li R. Gut microbiota involved in desulfation of sulfated progesterone metabolites: a potential regulation pathway of maternal bile acid homeostasis during pregnancy. Front Microbiol. (2022) 13:1023623. doi: 10.3389/fmicb.2022.1023623, PMID: 36338075 PMC9631449

[28] Diaz-Garcia H Vilchis-Gil J Castro-Cerritos KV Rivera-Susunaga E Klünder-Klünder M Granados-Riveron T. Association between maternal diet, smoking, and the placenta MTHFR 677C/T genotype and global placental DNA methylation. Placenta. (2024) 146:17–24. doi: 10.1016/j.placenta.2023.12.017, PMID: 38160599

[29] Kaczmarek MM Najmula J Guzewska MM Przygrodzka E. MiRNAs in the Peri-implantation period: contribution to embryo-maternal communication in pigs. Int J Mol Sci. (2020) 21:2229. doi: 10.3390/ijms21062229, PMID: 32210170 PMC7139304

[30] Del Gobbo GF Konwar C Robinson WP. The significance of the placental genome and methylome in fetal and maternal health. Hum Genet. (2020) 139:1183–96. doi: 10.1007/s00439-019-02058-w, PMID: 31555906 PMC7093232

[31] Lamy E Mau M. Saliva proteomics as an emerging, non-invasive tool to study livestock physiology, nutrition and diseases. J Proteome. (2012) 75:4251–8. doi: 10.1016/j.jprot.2012.05.007, PMID: 22583933

[32] López-Martínez MJ Lamy E Cerón JJ Ayala I Contreras-Aguilar MD Henriksen IH. Changes in the saliva proteome analysed by gel-proteomics in horses diagnosed with equine gastric ulcer syndrome (EGUS) at diagnosis and after successful treatment. Res Vet Sci. (2024) 167:105112. doi: 10.1016/j.rvsc.2023.105112, PMID: 38176208

[33] Pavithran S Murugan M Mannu J Yogendra K Balasubramani V Sanivarapu H. Identification of salivary proteins of the cowpea aphid *Aphis craccivora* by transcriptome and LC-MS/MS analyses. Insect Biochem Mol Biol. (2024) 165:104060. doi: 10.1016/j.ibmb.2023.104060, PMID: 38123026

